# Dynamical modeling of uncertain interaction-based genomic networks

**DOI:** 10.1186/1471-2105-16-S13-S3

**Published:** 2015-09-25

**Authors:** Daniel N Mohsenizadeh, Jianping Hua, Michael Bittner, Edward R Dougherty

**Affiliations:** 1 Department of Statistics, Texas A&M University, College Station, TX 77843, USA; 2 Department of Electrical and Computer Engineering, Texas A&M University, College Station, TX 77843, USA; 3 Center for Bioinformatics and Genomic Systems Engineering, Texas A&M University, College Station, TX 77843, USA

**Keywords:** Dynamical model, Uncertain networks, Algorithm design

## Abstract

**Background:**

Most dynamical models for genomic networks are built upon two current methodologies, one process-based and the other based on Boolean-type networks. Both are problematic when it comes to experimental design purposes in the laboratory. The first approach requires a comprehensive knowledge of the parameters involved in all biological processes *a priori*, whereas the results from the second method may not have a biological correspondence and thus cannot be tested in the laboratory. Moreover, the current methods cannot readily utilize existing curated knowledge databases and do not consider uncertainty in the knowledge. Therefore, a new methodology is needed that can generate a dynamical model based on available biological data, assuming uncertainty, while the results from experimental design can be examined in the laboratory.

**Results:**

We propose a new methodology for dynamical modeling of genomic networks that can utilize the interaction knowledge provided in public databases. The model assigns discrete states for physical entities, sets priorities among interactions based on information provided in the database, and updates each interaction based on associated node states. Whenever uncertainty in dynamics arises, it explores all possible outcomes. By using the proposed model, biologists can study regulation networks that are too complex for manual analysis.

**Conclusions:**

The proposed approach can be effectively used for constructing dynamical models of interaction-based genomic networks without requiring a complete knowledge of all parameters affecting the network dynamics, and thus based on a small set of available data.

## Background

Cellular regulatory networks consist of thousands of molecular processes/reactions collected over years from many studies. Based on their relationships and functions, these interactions and their associated molecular entities have been categorized into various pathways. Biologists have long used pathway knowledge to help derive the dynamics of cellular interactions, generate plausible hypotheses, and design promising experiments; however, our knowledge of regulatory networks is growing quickly and manual exploration is becoming increasingly difficult. The challenge of understanding network dynamics has become overwhelming in cancer drug development because multiple pathways are commonly involved and the direct targets of drug intervention are often multiple steps away from the key mechanism to be controlled, which could be in a different compartment and different pathway. Thus, a salient goal of computational biology is to simulate the dynamics of genetic/molecular regulatory networks based on available biological knowledge and in doing so help biologists generate meaningful hypotheses and improve experimental design.

A considerable amount of pathway knowledge centered around biological interactions is currently being curated into well organized public databases, such as PID [1], Reactome [2,3], and Pathway Commons [4], where the interactions and their associated physical entities are defined and shared via a standard language such as BioPAX [5]. The popular standard, Systems Biology Markup Language (SBML), has adopted systems of Ordinary Differential Equations (ODEs) or Partial Differential Equations (PDEs) as a natural way to describe the fine-grained interactions inside the pathways [6]. Along with the emergence of SBML, there are softwares that can run ODE and/or PDE-based cell models, e.g. LibSBML [7], CellDesigner [8], and the Virtual Cell [9]. However, such an approach demands complete information on the rate equations and their parameters in all reactions to be determined before any simulation can be conducted [10]. This requirement is commonly not available in translational research, for instance, in drug discovery [11].

Taking a coarse perspective, qualitative methods such as Boolean networks for regulatory networks [12] or Petri net for metabolic networks [13], focus on the physical entities rather than interactions. In these methods genes and gene products are linked by logical or algebraic relationships. While there are some popular knowledge databases, such as KEGG [14], where direct relationships are readily available, these cannot be directly adapted to interaction-centered databases (KEGG allows both relation and reaction, although relations are commonly provided.). Although it is possible to manually convert such databases into a form fit for Boolean modeling, the translation is not unique and can be unstable in relation to small interaction alterations. There are also software tools that can be used to run such simulations, including CellNetAnalyzer [15], CellNOpt [16], BooleanNet [17], and CPN tools [18].

Furthermore, all the existing approaches, differential equations, Boolean, or Petri net, require a complete and exact knowledge of the network, including the networks readily available from the public databases, whereas such knowledge associated with the biological processes is often incomplete and admits multiple possibilities. Although biologists routinely take these uncertainties into their own consideration, current computational approaches do not. Hence, a huge amount of curated pathway knowledge cannot be effectively utilized by the computer.

To help remedy this situation, we proposed a general scheme in which uncertainty is considered as part of the system and all possible outcomes that are attainable due to different possible ways in which pathway steps can be carried out will be considered accordingly. Via this approach, one can apply knowledge retrieved from interaction-centered knowledge databases directly to perform systematic simulations. Ultimately, with more meaningful predictions of network dynamics, a better guide to experimentation can be achieved.

This paper is organized as follows. In the Methods section, we provide some definitions and introduce an interaction-based network model which can be generated from an input biological database. We discuss about the dynamic trajectories of a network and the concept of uncertainty in network dynamics. An abstract form of the dynamical modeling algorithm for computing the dynamic trajectories characterizing the dynamical behavior of a network for a given initial condition is also provided in this section. A study on the dynamics of a number of real data networks using our dynamical modeling methodology is given in the Results and discussion section. We also describe our current research activities. Finally, we provide our concluding remarks in the Conclusions section.

## Methods

To produce our network model, we employ knowledge retrieved from public knowledge databases such as PID and Reactome. To simplify the discussion, we assume that knowledge is shared via the standard language BioPAX, specifically, BioPAX3. To facilitate efficient simulation, we first represent the interactions and associated physical entities shared in BioPAX using a set of nodes and edges.

### An interaction-based network model

The model *M*, of the biological database under study, is a 2-tuple *M *= (*N, E*), where *N *is the set of network nodes representing the biological entities in the database, including genes, proteins, complexes, molecules, transcripts, metabolites and chemicals, interacting with each other, and *E *is the set of network hyper-edges (a hyper-edge is an edge that can have multiple inputs/outputs), or simply edges, representing the biological interactions and processes in the database. The set of network nodes *N *and network edges *E *can be automatically generated from a simple text-based input file describing the biological interactions using our developed software package [19]. A detailed description on preparing the input file can be found in the Appendix section.

Each node in the network has a *value*, where the abstract term "value" is interpreted based on the characteristics of that biological entity. It may represent the concentration level of a protein/chemical or the expression level of a gene, indicating whether the gene is active or inactive. It does not necessarily indicate the relationship, such as concentration relationship, between different biological entities. The concentration of proteins, chemicals, and so on, in the cell are continuous variables; however, there are reasons why discrete variables are more suitable. First, accurate real-time concentration measurements are almost impossible. Second, a gene/protein typically participates in a biological process if its concentration is above a threshold level and such behavior is readily handled using discrete variables. Finally, from a control system engineering perspective, it is computationally and practically easier to deal with discrete-time systems rather than continuous-time systems. Therefore, we assume that network nodes take non-negative integer values. The state of a network with *m *nodes, namely *N *= {*n*_1_, *n*_2_,..., *n_m_*}, is a vector $x\in {\mathbb{Z}}_{\geq 0}^{m},{\mathbb{Z}}_{\geq 0}$ being the set of non-negative integers, where each element of *x *is the value of a node *n ∈ N*. Let us also denote by *y *the vector of observable/measurable states, representing the subset of genes, proteins, transcripts, and so on, whose activities can be observed or measured and are of our interest. We represent the *i*-th node in *N *by *n_i_*. The same notation will be used for network edges, for example, *e_j _*refers to the *j*-th edge in *E*.

Each network edge represents a corresponding biological process or interaction in which, generally, the process reactants (inputs) interact with each other to form products (outputs) under a set of conditions. Thus, in general, each edge will have *input *and *output *nodes. In cases where a process is controlled, for instance, via activation or inhibition by a biological entity, a corresponding *control *node is associated to that edge. The input, output, and control nodes of the *i*-th edge are denoted by *e_i_.I, e_i_.O *and *e_i_.C*, respectively. We assume that an edge (a process) can dynamically take place if all its input and activator nodes have nonzero values and its inhibitor nodes have zero values. We use the following rules to update the node values when the *i*-th edge is being dynamically processed:

(1) $$\begin{array}{c} e_{i\cdot }I{|}_{\text{new}}=e_{i\cdot }I{|}_{\text{old}}-1\\ e_{i\cdot }O{|}_{\text{new}}=e_{i\cdot }O{|}_{\text{old}}+1\\ e_{i\cdot }C{|}_{\text{new}}=e_{i\cdot }C{|}_{\text{old}} \end{array}$$

Arrows indicate the directions of processes/edges graphically. A dashed arrow is used to show an activation or inhibition in a network process. As an example, a process in which protein *A *converts to protein *B *if the activator protein *C *presents is shown as follows:

(2) $$\begin{array}{ccc} & C & \\ & ⇣ & \\ A & \to & B \end{array}$$

To generate a network model for the above process, we define the set of network nodes by *N *= {*n*_1_, *n*_2_, *n*_3_} = {*A, B, C*}, and the set of network edges by *E *= {*e*_1_}:

(3) $$\begin{array}{ccc} & n_{3} & \\ & ⇣ & \\ n_{1} & \to & n_{2}\\ & e_{1} & \end{array}$$

where *n*_1_, *n*_2 _and *n*_3 _are the input, output and activator nodes of *e*_1_, respectively. Supposing that the initial condition is given by *x*_0 _= [1, 0, 1]*^T^*, since the input and activator nodes have nonzero values, the updated state vector, following the rules in (1), becomes *x*_1 _= [0, 1, 1]*^T^*, and we write:

(4) $$\underset{x_{0}}{\underset{︸}{\left[\begin{array}{c} 1\\ 0\\ 1 \end{array}\right]}}\overset{e_{1}}{\underset{}{\to }}\underset{x1}{\underset{︸}{\left[\begin{array}{c} 0\\ 1\\ 1 \end{array}\right]}}$$

Generally, in conversion processes to which activator entities are associated, if the activator is removed, the conversion will be halted and even reversed. Also, as can be seen in real cellular systems, if the activator does not present then the new input species coming from other processes will not convert to outputs and will accumulate; thus, the outputs gradually degrade and will be depleted. Based on this observation, we make the following assumption.

**Assumption 1 ***If in a conversion process, the activator entity has zero value and the outputs (right-hand side entities) have nonzero values, then the process takes place in the reverse direction*.

Considering the statement of Assumption 1 for any conversion process in the network under study to which an activator entity is associated, an additional network edge corresponding to the reverse direction of the original process will be automatically added to *E*. For instance, for the conversion process in (2), we present the network model in the following form:

(5) $$\begin{array}{ccc} & n_{3} & \\ & ⇣ & \\ n_{1} & \overset{}{\underset{e_{1}\left(\text{rev}:\;e_{2}\right)}{\to }} & n_{2} \end{array}$$

Now, if *x*_0 _= [0, 1, 0]*^T^*, then the process takes place in the reverse direction resulting in *x*_1 _= [1, 0, 0]*^T ^*.

For the set of biological processes in which a transcription takes place we follow the BioPAX format in which no input is assigned, but only an activator and an output are defined, these representing whether the transcription has occurred or not. Thus, for this subset of network edges we use the following logic to update the output node value:

(6) $$\begin{array}{cccc} \text{ if} & e_{i\cdot }C_{act}>0 & \text{then} & {e_{i}}_{\cdot }O{|}_{\text{new}}=1\\ \text{else if} & e_{i\cdot }C_{act}=0 & \text{then} & {e_{i}}_{\cdot }O{|}_{\text{new}}=0 \end{array}$$

where *e*_*i*_.*C*_*act *_represents the activator node associated to *e_i_*. Once we have updated the output node of a transcription process we will not update that node any further until the activator node value changes. For example, suppose that transcription *B *happens if protein *A *presents. We thus define *N *= {*n*_1_, *n*_2_} = {*A, B*} and *E *= {*e*_1_}:

(7) $$e_{1}:n_{1}⇢n_{2}$$

Now, for the initial condition *x*_0 _= [1, 0]*^T ^*, the updated state vector will be *x*_1 _= [1, 1]*^T ^*, implying that the corresponding transcription happens.

### Dynamic trajectories and uncertainty in network dynamics

Once an interaction-based network model *M *is developed, a dynamical analysis can be performed. Given a network model *M *= (*N, E*), an initial condition vector *x*_0_, and defining the parameter *time step*, the network state vector *x *can be updated at each time step based on our proposed algorithm, presented later in this section. The abstract term *time step *does not necessarily reflect the real clock time that biological processes take to complete; the real clock time for one time step can be substantially different from another time step. We denote by *x_k _*the network state vector at the *k*-th time step. Generally, a complete knowledge of all parameters affecting the dynamics of a biological process is not available. Besides, in most practical cases, these parameters can not be even measured or observed. Therefore, in general, for a given network initial condition *x*_0_, different sequences of processes can happen, these being referred to as *dynamic trajectories *and denoted by *T*. Each trajectory *t *∈ *T*, indicates one possible sequence of processes that can take place. We describe the trajectory *t ∈ T *by *t *= {*e_i_, e_j _*,...}, for appropriate values of *i, j*,..., which means that in the first time step process *i *(edge *e_i_*) happens, in the second time step process *j *(edge *e_j_*) takes place, and so on. The fact that, for a given *x*_0_, multiple dynamic trajectories can be computed reflects uncertainty that we refer to as *dynamics uncertainty*. In cases where a possibly limited knowledge about the biological processes is available, it can be effectively used to reduce the dynamics uncertainty of the model. This can be accomplished by assigning appropriate properties to the set of network edges, as discussed next. We emphasize that the dynamic trajectories of a network depend on the properties assigned to the network edges as well as the network initial condition.

### Network edge properties

To each network edge a set of properties, upon availability of corresponding information in the biological database under study, can be associated, these being critical in characterizing network dynamics. For each network edge, these properties will be stored in a vector associated with that specific edge and can be accessed in the dynamical modeling algorithm, to be discussed shortly. For instance, to access the property *X *of the *i*-th edge we call *e_i_.X*. The dynamical modeling algorithm consists of conditional statements being functions of the edge properties; thus, the algorithm outputs depend on the edge properties. The properties, *speed *and *priority*, are assigned to each edge according to the nature or earlier measurements of that biological process.

#### Speed

The *speed *label describes the time scale needed for a process to complete [20,21]. This is critical in dynamical network modeling because fast processes, such as complex assembly processes in which a group of proteins bind together to form a complex, happen almost instantaneously, while slow processes, such as transcription and apoptosis (cell death), take much longer times to complete. If such qualitative knowledge on the time scale of processes can be obtained from the biological database, then corresponding speed labels can be assigned to network edges, thereby reducing uncertainty in the dynamical model; otherwise, the dynamical model will embrace such uncertainty. In BioPAX3, processes are categorized into Conversion, Template Reaction, Control, Molecular Interaction, and Genetic Interaction. In process-based knowledge databases, almost all processes belong to the first three categories and every Control process is essentially associated with either a Conversion or a Template Reaction process. By default, to any process labeled as Conversion in the knowledge database, we assign a "fast" label; however, for those labeled as Template Reaction, such as processes that end in a phenotypical or pathway level event, a "slow" label is assigned, because the Control and Conversion interactions generally happen considerably faster than the Template Reaction processes. If knowledge about the time scale of a process is not available, then a "fast" label is assigned to the corresponding edge by default.

To show how the speed property can affect network dynamics, consider proteins *A*, *B*, and *C *interacting according to:

(8) $$\begin{array}{c} A+B\to A.B\\ C+A.B\to A+B.C\\ A.B\to \text{Apoptosis} \end{array}$$

where the first two processes are of Conversion (complex assembly) type and the last one, resulting in apoptosis, is a Template Reaction process. We define *N *= {*n*_1_, *n*_2_, *n*_3_, *n*_4_, *n*_5_, *n*_6_} = {*A*, *B*, *C*, *A.B*, *B.C*, Apoptosis}, and *E *= {*e*_1_, *e*_2_, *e*_3_}:

(9) $$\begin{array}{ccc} n_{1}+n_{2} & \overset{e_{1}}{\underset{}{\to }} & n_{4}\;\;\;\;\;\;\\ n_{3}+n_{4} & \overset{e_{2}}{\underset{}{\to }} & n_{1}+n_{5}\\ n_{4} & \overset{e_{3}}{\underset{}{\to }} & n_{6}\;\;\;\;\;\; \end{array}$$

Also, suppose that the initial condition is given as *x*_0 _= [1, 1, 1, 0, 0, 0]*^T ^*. According to the above description of processes, *e*_1 _and *e*_2 _happen fast, while *e*_3 _takes more time to complete. If such time-scale knowledge is not incorporated into the network model in the form of appropriate edge properties and we, by default, assign a "fast" label to all the edges, then the network state vector can be updated in two different ways, as detailed below, thereby introducing uncertainty to the dynamical model:

(10) $$\underset{x_{0}}{\underset{︸}{\left[\begin{array}{c} 1\\ 1\\ 1\\ 0\\ 0\\ 0 \end{array}\right]}}\overset{e_{1}}{\underset{}{\to }}\underset{x_{1}^{1}}{\underset{︸}{\left[\begin{array}{c} 0\\ 0\\ 1\\ 1\\ 0\\ 0 \end{array}\right]}}\overset{e_{2}}{\underset{}{\to }}\underset{x_{2}^{1}}{\underset{︸}{\left[\begin{array}{c} 1\\ 0\\ 0\\ 0\\ 1\\ 0 \end{array}\right]}}$$

(11) $$\underset{x_{0}}{\underset{︸}{\left[\begin{array}{c} 1\\ 1\\ 1\\ 0\\ 0\\ 0 \end{array}\right]}}\overset{e_{1}}{\underset{}{\to }}\underset{x_{1}^{1}}{\underset{︸}{\left[\begin{array}{c} 0\\ 0\\ 1\\ 1\\ 0\\ 0 \end{array}\right]}}\overset{e_{3}}{\underset{}{\to }}\underset{x_{2}^{2}}{\underset{︸}{\left[\begin{array}{c} 0\\ 0\\ 1\\ 0\\ 0\\ 1 \end{array}\right]}}$$

In the above updates of the network states, $x_{k}^{p}$ indicates the *p*-th possible update of the state vector at the *k*-th time step. Now, if one applies the process timescale knowledge by assigning a "fast" label to the speed of *e*_1 _and *e*_2_, and a "slow" label to that of *e*_3_, then the state vector will be updated only according to the sequence in (10). Use of time-scale information reduces uncertainty in the dynamical model.

#### Priority

The *priority *label helps when there exist two or more processes with nonzero input and activator nodes, and zero inhibitor nodes, meaning that they have equal probability to happen; however, a prior knowledge states that only one or few of them will happen in practice. Priority is usually not provided in the knowledge database; but in many cases we can set default priorities based on experience. For instance, if an entity is an input in process 1 and a control node in process 2, then process 1 has higher priority than process 2, because in process 1, the entity will be converted and not be available for control. For example, PIP3 is needed to phosphorylate AKT, yet PIP3 can also be converted to PIP2 by PTEN. Hence, if PTEN exists, PIP3 will be converted and not be able to phosphorylate AKT. Therefore, upon availability of such prior knowledge, one can assign priority labels to the network edges and reduce the uncertainty in the dynamical model; if this is not the case, then no priority labels will be assigned and the dynamical model will contain all of the possibilities. Now, consider the following example in which proteins *A*, *B*, *C*, and *D *bind together to form complex assemblies:

(12) $$\begin{array}{c} A+B\to A.B\\ C+A.B\to A+B.C\\ D+A.B\to B+A.D \end{array}$$

We define *N *= {*n*_1_, *n*_2_, *n*_3_, *n*_4_, *n*_5_, *n*_6_, *n*_7_} = {*A, B, C, D, A.B, B.C, A.D*} and *E *= {*e*_1_, *e*_2_, *e*_3_}:

(13) $$\begin{array}{c} {\text{n}}_{\text{1}}\text{ + }{\text{n}}_{\text{2}}\overset{{\text{e}}_{\text{1}}}{\underset{}{\to }}{\text{n}}_{\text{5}}\\ {\text{n}}_{\text{3}}\text{ + }{\text{n}}_{\text{5}}\overset{{\text{e}}_{\text{2}}}{\underset{}{\to }}{\text{n}}_{\text{1}}\text{ + }{\text{n}}_{\text{6}}\\ {\text{n}}_{\text{4}}\text{ + }{\text{n}}_{\text{5}}\overset{{\text{e}}_{\text{3}}}{\underset{}{\to }}{\text{n}}_{\text{2}}\text{ + }{\text{n}}_{\text{7}} \end{array}$$

Since all the above processes are of Conversion type, we assign the "fast" label to the speed property of all the edges. Assuming that the initial condition is given by *x*_0 _= [1, 1, 1, 1, 0, 0, 0]*^T ^*, the network state vector can be updated in two different ways:

(14) $$\underset{x_{0}}{\underset{︸}{\left[\begin{array}{c} 1\\ 1\\ 1\\ 1\\ 0\\ 0\\ 0 \end{array}\right]}}\overset{e_{1}}{\underset{}{\to }}\underset{x_{1}^{1}}{\underset{︸}{\left[\begin{array}{c} 0\\ 0\\ 1\\ 1\\ 1\\ 0\\ 0 \end{array}\right]}}\overset{e_{2}}{\underset{}{\to }}\underset{x_{2}^{1}}{\underset{︸}{\left[\begin{array}{c} 1\\ 0\\ 0\\ 1\\ 0\\ 1\\ 0 \end{array}\right]}}$$

(15) $$\underset{x_{0}}{\underset{︸}{\left[\begin{array}{c} 1\\ 1\\ 1\\ 1\\ 0\\ 0\\ 0 \end{array}\right]}}\overset{e_{1}}{\underset{}{\to }}\underset{x_{1}^{1}}{\underset{︸}{\left[\begin{array}{c} 0\\ 0\\ 1\\ 1\\ 1\\ 0\\ 0 \end{array}\right]}}\overset{e_{3}}{\underset{}{\to }}\underset{x_{2}^{2}}{\underset{︸}{\left[\begin{array}{c} 0\\ 1\\ 1\\ 0\\ 0\\ 0\\ 1 \end{array}\right]}}$$

Now, suppose that a prior knowledge indicates that, in situations where *e*_2 _and *e*_3 _can mathematically happen at the same time owing to nonzero input nodes, *e*_2 _has higher priority than *e*_3 _to take place. Accommodating this priority information into our network model by assigning the label "$e_{2}^{prio}>e_{3}^{prio}$" to the priority of *e*_2_, and "$e_{3}^{prio}<e_{2}^{prio}$" to that of *e_3 _*, the state vector can now be updated based on the sequence of processes given in (14).

### Dynamical modeling algorithm

An abstract form of the algorithm proposed to perform the dynamical analysis is provided below. A detailed algorithm can be found in the Additional file 1 to this manuscript. The algorithm is based on the breadthfirst search algorithm [22]. It takes the network model *M *= (*N, E*) and an initial condition *x*_0 _as inputs, and return the resulting dynamic trajectories *T*, and the corresponding updates of the state vector *x_k _*at each time step *k*. There are two approaches to update the network state vector: synchronous and asynchronous [23,17]. With synchronicity, multiple network edges can be processed at the same time step; while with asynchronicity, only one network edge is processed at each time step. Our software package [19] has the capability to update the network state vector using either approach. Since, in general, the asynchronous approach yields a larger set of possible dynamic trajectories compared to the synchronous method [24], asynchronicity is the default method to update the network state vector. The abstract algorithm given below updates the state vector asynchronously.

**Algorithm **Dynamical Modeling Algorithm

**Inputs: **(*N, E, x*_0 _)

**Outputs: **(*T*, *x*)

1: *k *← 1, *T *← ∅, *x_in _*← *x*_0_, *S_in_*, *x_k_*, *S_k _*← ∅

2: run States Update Subroutine (Inputs: (*x_in_*, *S_in_*, *x_k_*, *S_k_*, *T*), Outputs: (*x_k_*, *S_k_*, *T *))

3: **while ***x_k _*≠ ∅ **do**

4:   new time step: *k *← *k *+ 1, *x_k_*, *S_k _*← ∅

5:   **for **each updated state vector $x_{k-\text{1}}^{i}$ in *x*_*k *- 1 _**do**

6:      **if **corresponding $S_{k-\text{1}}^{i}$ forms a cycle **then**

7:         if $S_{k-\text{1}}^{i}\notin T$, then add $S_{k-\text{1}}^{i}$ to *T*

8:      **else**

9:         $x_{in}\leftarrow x_{k-1}^{i},S_{in}\leftarrow S_{k-1}^{i}$

10:         run States Update Subroutine (Inputs: (*x*_*in*_, *S_in_*, *x_k_*, *S_k_*, *T *), Outputs: (*x_k_, S_k_, T *))

11:      **end if**

12:   **end for**

13: **end while**

States Update Subroutine

**Inputs: **(*x*_*in*_, *S_in_*, *x_k_*, *S_k_*, *T*)

**Outputs: **(*x*_*k*_, *S*_*k*_, *T*)

1: initialize all nodes by *x*_*in*_

2: *E_v _*← find all edges that have nonzero input nodes, nonzero activator nodes and zero inhibitor nodes (if applicable)

3: **if ***E_v _*≠ ∅ **then**

4:   remove edges in *E_v _*having relatively slow/low priority label

5:   **for **each edge in *E_v _***do**

6:      initialize all nodes by *x*_*in*_

7:      $x_{k}^{new}$ ← update the state vector using the given rules

8:      *x*_*k *_← {*x*_*k*_, $x_{k}^{new}$}

9:      $S_{k}^{new}$ ← the sequence of edges yielded $x_{k}^{new}$

10:      $S_{k}\leftarrow \left\{S_{k},S_{k}^{new}\right\}$

11:   **end for**

12: **else**

13:   if *S*_*in *_∉ *T*, then add *S*_*in *_to *T*

14: **end if**

**Figure 1:**
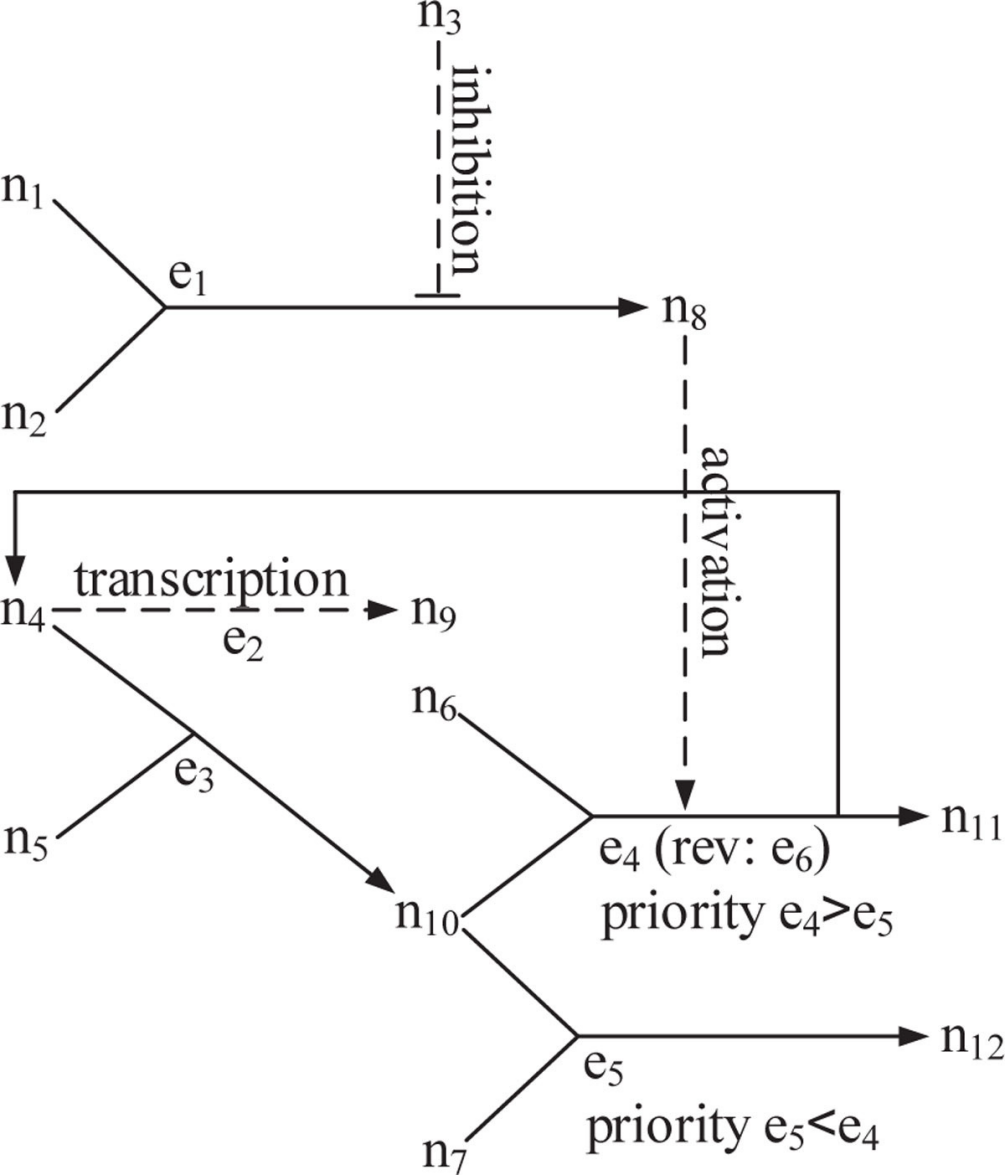
**An illustrative network**.

To illustrate the algorithm, consider the network in Figure 1, where *N *= {*n*_1_, *n*_2_,..., *n*_12_} and *E *= {*e*_1_, *e*_2_,..., *e*_6_}. Suppose that the biological database provides the following information about the processes in this network: in process/edge *e*_1 _proteins *n*_1 _and *n*_2 _bind together to form the complex *n*_8_, if the inhibitor gene *n*_3 _does not express; *e*_2 _is a transcription process (the only slow process in this example) in which *n*_4 _is the activator and *n*_9 _accounts for the status of the transcription; in *e*_3 _proteins *n*_4 _and *n*_5 _convert to complex *n*_10 _; in *e*_4 _proteins *n*_6 _and *n*_10 _form compound *n*_11 _and release *n*_4_, if the activator *n*_8 _presents; and finally, in *e*_5 _proteins *n*_7 _and *n*_10 _convert to compound *n*_12_, but this process is assumed to have less priority compared to *e*_4 _. Based on the above description and according to the statement of Assumption 1, our method automatically adds edge *e*_6 _to the set of network edges to account for the reverse direction of *e*_4_. We incorporate the above biological information into the network model by assigning appropriate labels to the properties of network edges (see Table 1). Suppose that the initial condition is:

**Table 1:** Edge properties for the illustrative network.

label\edge	*e*_1_	*e*_2 _	*e*_3 _	*e*_4 _	*e*_5 _	*e*_6 _
speed	fast	slow	fast	fast	fast	fast
priority				$e_{4}^{prio}>e_{5}^{prio}$	$e_{5}^{prio}>e_{4}^{prio}$	

$x_{0}={\left[1,1,0,1,1,1,1,0,0,0,0,0\right]}^{T}$

Following the proposed algorithm to calculate the dynamic trajectories and state vector updates, we get (the sign ▷ is used for comments, and "SU" indicates the States Update Subroutine):

- line 1: *k *← 1, *T *← ∅, *x_in _*← *x*_0_, *S_in_*, *x*_1_, *S*_1 _← ∅

- line 2: run States Update Subroutine

- line SU1: initialize all nodes by *x_in_*

- line SU2: *E_υ_*= {*e*_1_, *e*_2_, *e*_3_}

- line SU4: *E_υ_*= {*e*_1_, *e*_3_} ⊳ *e*_2 _is removed because it is slower than other edges

- line SU6: initialize all nodes by *x_in_*

- line SU7-8: $x_{1}^{new}=x_{1}^{1}={\left[0,0,0,1,1,1,1,1,0,0,0,0\right]}^{T},x_{1}\leftarrow x_{1}^{new}$

- line SU9-10: $S_{1}^{new}=S_{1}^{1}=\left\{e_{1}\right\},S_{1}\leftarrow S_{1}^{new}$

- line SU6: initialize all nodes by *x_in_*

- line SU7-8: $x_{1}^{new}=x_{1}^{2}={\left[\text{1},\text{1},0,0,0,\text{1},\text{1},0,0,\text{1},0,0\right]}^{T},x_{1}\leftarrow \left\{x_{1},x_{1}^{new}\right\}$

- line SU9-10: $S_{1}^{new}=S_{1}^{2}=\left\{e_{3}\right\},S_{1}\leftarrow \left\{S_{1},S_{1}^{new}\right\}$

- line 4: *k *← 2, *x*_2_, *S*_2 _← ∅

- line 9: $x_{in}\leftarrow x_{1}^{1},S_{in}\leftarrow S_{1}^{1}$

- line 10: run States Update Subroutine

- line SU1: initialize all nodes by *x_in_*

- line SU2: *E_υ_*= {*e*_2_, *e*_3_}

- line SU4: *E_υ_*= {*e*_3_}

- line SU6: initialize all nodes by *x_in_*

- line SU7-8: $x_{2}^{new}=x_{2}^{1}={\left[0,0,0,0,0,1,1,1,0,1,0,0\right]}^{T},x_{2}\leftarrow x_{2}^{new}$

- line SU9-10: $S_{2}^{new}=S_{2}^{1}=\left\{e_{1},e_{3}\right\},S_{2}\leftarrow S_{2}^{new}$

- line 9: $x_{in}\leftarrow x_{1}^{2},S_{in}\leftarrow S_{1}^{2}$

- line 10: run States Update Subroutine

- line SU1: initialize all nodes by *x_in_*

- line SU2: *E_υ _*= {*e*_1_, *e*_5_} ⊳ since *e*_4_, which has higher priority than *e*_5_, is not in *E_υ_*, then *e*_5 _will not be removed once line SU4 is processed.

- line SU6: initialize all nodes by *x_in_*

- line SU7-8: $x_{2}^{new}=x_{2}^{2}={\left[0,0,0,0,0,1,1,1,0,1,0,0\right]}^{T},x_{2}\leftarrow \left\{x_{2},x_{2}^{new}\right\}$

- line SU9-10: $S_{2}^{new}=S_{2}^{2}=\left\{e_{3},e_{1}\right\},S_{2}\leftarrow \left\{S_{2},S_{2}^{new}\right\}$

- line SU6: initialize all nodes by *x_in_*

- line SU7-8: $x_{2}^{new}=x_{2}^{3}={\left[\text{1},\text{1},0,0,0,\text{1},0,0,0,0,0,\text{1}\right]}^{T},x_{2}\leftarrow \left\{x_{2},x_{2}^{new}\right\}$

- line SU9-10: $S_{2}^{new}=S_{2}^{3}=\left\{e_{3},e_{5}\right\},S_{2}\leftarrow \left\{S_{2},S_{2}^{new}\right\}$

- line 4: *k *← 3, *x*_3_, *S*_3 _← ∅

- The next time steps can be followed easily.

Finally, the algorithm outputs are:

Dynamic trajectory *t*_1 _∈ *T *: $t_{\text{1}}:=S_{3}^{3}=\left\{e_{\text{3}},e_{\text{5}},e_{\text{1}}\right\}$

State updates for *t*_1 _∈ *T*: $x_{1}^{2},x_{2}^{3},x_{3}^{3}$

Dynamic trajectory *t*_2 _∈ *T*: $t_{2}:=S_{4}^{1}=\left\{e_{1},e_{3},e_{4},e_{2}\right\}$

State updates for *t*_2 _∈ *T*: $x_{1}^{1},x_{2}^{1},x_{3}^{1},x_{4}^{1}$

## Results and discussion

In this section we study the dynamics of 3 networks: BAK1-MCL1 interaction, the MAP Kinase pathway, and the ErbB2/ErbB3 pathway. For each network we calculate the dynamic trajectories for a given initial condition vector. For illustration, these networks are graphically plotted in Microsoft Visio software and provided here. The computer used to perform dynamical analysis and compute dynamic trajectories is a Sony VAIO laptop with Intel Core 2 Duo 1.67 GHz CPU and 2 GB RAM.

### BAK1-MCL1 interaction

#### Biological database and network model

**Figure 2:**
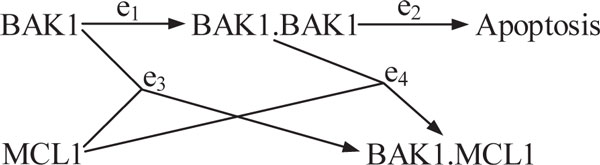
**BAK1-MCL1 interaction**.

Consider the network of BAK1-MCL1 interaction shown in Figure 2. In cells, once synthesized, protein BAK1 is localized at the mitochondria. Following the apoptotic stimulus, BAK1 will dimerize to form homodimers. These dimers will further oligomerize and trigger mitochondrial outer membrane permeabilization (MOMP) and induce apoptosis [25]. To counter such apoptosis stimulus and keep the the cell alive, an anti-apoptotic protein like MCL1 will bind to BAK1 with higher affinity and block BAK1 from forming homodimers, thereby stopping apoptosis [26]. Thus, in cancer cells, especially melanoma, MCL1 is commonly found to be overexpressed [27].

In this network X.Y indicates a binder complex produced by X and Y. We define the set of network nodes and edges by *N *= {*n*_1_, *n*_2_,..., *n*_5_} = {BAK1, MCL1, BAK1.BAK1, Apoptosis, MCL1.BAK1} and *E *= {*e*_1_, *e*_2_, *e*_3_, *e*_4_}. Process *e*_2 _is the only slow process in this network resulting in apoptosis; thus, we assign a "slow" label to the speed property of *e*_2 _and, by default, a "fast" label to *e*_1_, *e*_3_, and *e*_4 _. Also, as discussed, MCL1 binds to BAK1 with higher affinity than BAK1 binding to itself to form BAK1.BAK1 [26]; therefore, *e*_3 _has higher priority than *e*_1_, and we accordingly assign the priority label "$e_{1}^{prio}<e_{3}^{prio}$" to *e*_1_, and "$e_{3}^{prio}<e_{1}^{prio}$ " to *e*_3 _. In this network the observable state is *y *= *n*_4 _= Apoptosis, since it can be checked under a microscope.

#### Dynamic trajectories

Suppose that the network initial state vector is given by *x*_0 _= [2, 1, 0, 0, 0]*^T ^*, biologically meaning that the concentration of BAK1 is considerably higher than MCL1, the concentration of MCL1 is enough to participate in the processes in Figure 2, and other entities do not exist or have considerably low concentrations. For this initial condition and the assigned edge properties, our algorithm returns the following possible updates of the network state vector (for notational simplicity we drop the superscripts used for the updated state vectors):

(16) $$\underset{x_{0}}{\underset{︸}{\left[\begin{array}{c} 2\\ 1\\ 0\\ 0\\ 0 \end{array}\right]}}\overset{e_{3}}{\underset{}{\to }}\underset{x_{1}}{\underset{︸}{\left[\begin{array}{c} 1\\ 0\\ 0\\ 0\\ 1 \end{array}\right]}}\overset{e_{1}}{\underset{}{\to }}\underset{x_{2}}{\underset{︸}{\left[\begin{array}{c} 0\\ 0\\ 1\\ 0\\ 1 \end{array}\right]}}\overset{e_{2}}{\underset{}{\to }}\underset{x_{3}}{\underset{︸}{\left[\begin{array}{c} 0\\ 0\\ 0\\ 1\\ 1 \end{array}\right]}}$$

The dynamic trajectory in (16) is

(17) $$T=\left\{t_{1}\right\}=\left\{e_{3},e_{1},e_{2}\right\}$$

The dynamic trajectory obtained here yields the final value of the observable state *y*, the apoptosis status, to be 1, which biologically means that apoptosis takes place. This result agrees with our expectation from a biological point of view because initially there was insufficient amount of MCL1 to bind to the higher amount of BAK1 to avoid apoptosis. The CPU time to calculate the dynamic trajectories for this network using the computer specifications described above was 0.1 sec.

### MAP Kinase pathway

#### Biological database and network model

**Figure 3:**
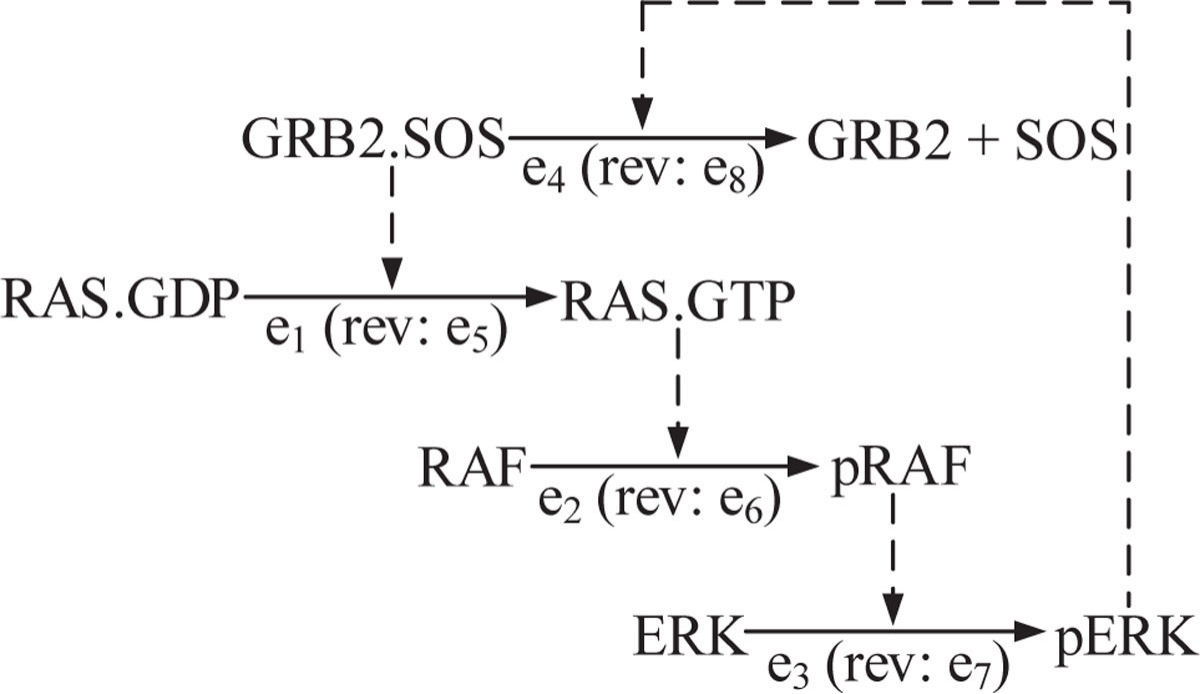
**MAP Kinase pathway**.

The dynamical study of pathways containing feedback processes has practical importance for experimental design and therapy. In this example we considered the set of biological processes shown in Figure 3, which is based on the well-studied MAK Kinase pathway. Under the stimulus of a growth factor, GRB2.SOS complex will be recruited to the cell membrane, which will transform the GDP-bound RAS into its active form, GTP-bound RAS. The activated RAS will in turn activate the Raf/MEK/ERK pathway [28]. However, it has been shown that the activation of RAS is often short and transient, due to a negative feedback loop through ERK, which dissociates the GRB2.SOS complex, thereby inducing an oscillatory pattern in ERK activity [29].

The set of network nodes is *N *= {*n*_1_, *n*_2_,..., *n_9_*} = {RAS.GDP, RAS.GTP, GRB2.SOS, RAF, pRAF, ERK, pERK, GRB2, SOS} and the network edges are *E *= {*e*_1_, *e*_2_,..., *e*_8 _}. The database provides information about the processes *e*_1_, *e*_2_, *e*_3_, and *e*_4 _. Owing to Assumption 1, our algorithm automatically generates the corresponding edges *e*_5_, *e*_6_, *e*_7_, and *e*_8 _to account for the reversible direction of the original processes. By default we assign a "fast" label to all edges. Since the database gives no information about the priority between the processes, no priority labels are assigned.

#### Dynamic trajectories

Given the initial condition *x*_0 _= [1, 0, 1, 1, 0, 1, 0, 0, 0]*^T ^*, our algorithm yields the following dynamic trajectory:

(18) $$T=\left\{t_{1}\right\}=\left\{e_{1},e_{2},e_{3},e_{4},e_{5},e_{6},e_{7},e_{8},...\right\}$$

and this sequence repeats. The effect of the feedback process can be seen in the dynamic trajectory as the sequence of edges is repeating over time. The network state vector evolves as follows:

(19) $$\underset{x_{0}}{\underset{︸}{\left[\begin{array}{c} 1\\ 0\\ 1\\ 1\\ 0\\ 1\\ 0\\ 0\\ 0 \end{array}\right]}}\overset{e_{1}}{\underset{}{\to }}\underset{x_{1}}{\underset{︸}{\left[\begin{array}{c} 0\\ 1\\ 1\\ 1\\ 0\\ 1\\ 0\\ 0\\ 0 \end{array}\right]}}\overset{e_{2}}{\underset{}{\to }}\underset{x_{2}}{\underset{︸}{\left[\begin{array}{c} 0\\ 1\\ 1\\ 0\\ 1\\ 1\\ 0\\ 0\\ 0 \end{array}\right]}}\overset{e_{3}}{\underset{}{\to }}\underset{x_{3}}{\underset{︸}{\left[\begin{array}{c} 0\\ 1\\ 1\\ 0\\ 1\\ 0\\ 1\\ 0\\ 0 \end{array}\right]}}\overset{e_{4}}{\underset{}{\to }}\underset{x_{4}}{\underset{︸}{\left[\begin{array}{c} 0\\ 1\\ 0\\ 0\\ 1\\ 0\\ 1\\ 1\\ 1 \end{array}\right]}}\overset{e_{5}}{\underset{}{\to }}\underset{x_{5}}{\underset{︸}{\left[\begin{array}{c} 1\\ 0\\ 0\\ 0\\ 1\\ 0\\ 1\\ 1\\ 1 \end{array}\right]}}\overset{e_{6}}{\underset{}{\to }}\underset{x_{6}}{\underset{︸}{\left[\begin{array}{c} 1\\ 0\\ 0\\ 1\\ 0\\ 0\\ 1\\ 1\\ 1 \end{array}\right]}}\overset{e_{7}}{\underset{}{\to }}\underset{x_{7}}{\underset{︸}{\left[\begin{array}{c} 1\\ 0\\ 0\\ 1\\ 0\\ 1\\ 0\\ 1\\ 1 \end{array}\right]}}\overset{e_{8}}{\underset{}{\to }}\underset{x_{0}}{\underset{︸}{\left[\begin{array}{c} 1\\ 0\\ 1\\ 1\\ 0\\ 1\\ 0\\ 0\\ 0 \end{array}\right]}}\overset{e_{1}}{\underset{}{\to }}.....$$

The CPU time for this network was 0.3 sec.

### ErbB2/ErbB3 pathway

#### Biological database and network model

**Figure 4:**
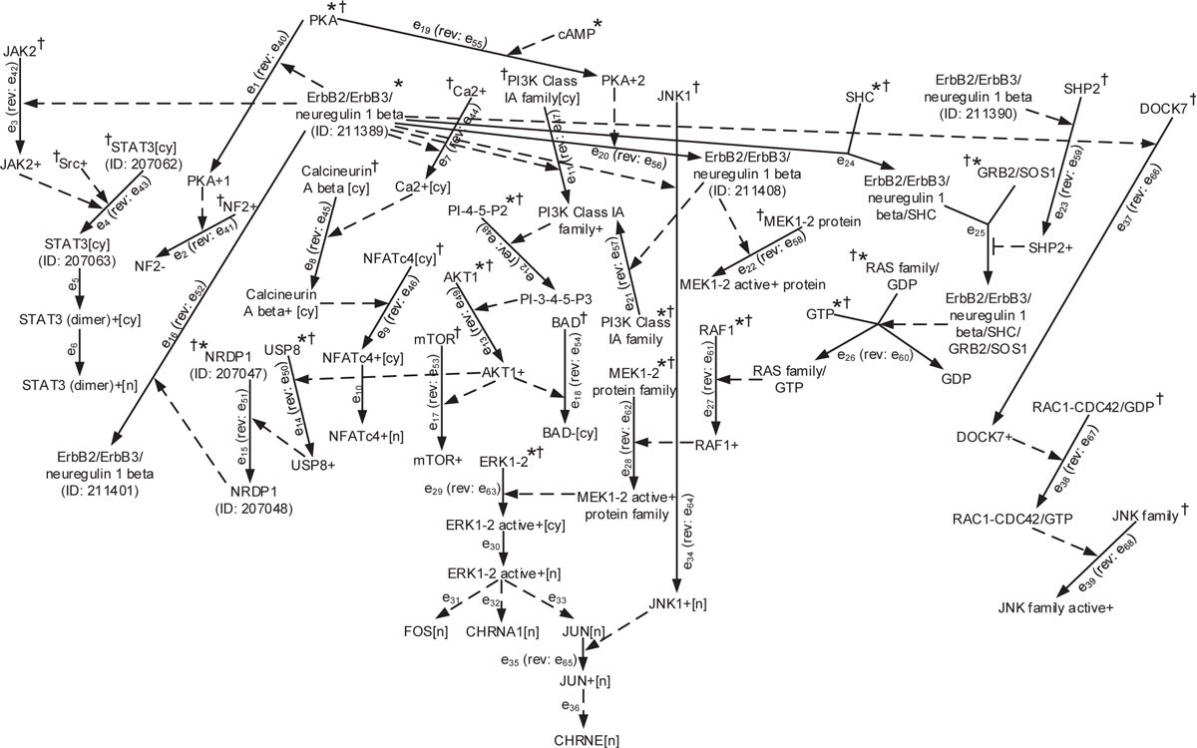
**ErbB2/ErbB3 pathway**.

In this example (see Figure 4) we studied the dynamics of the ErbB2/ErbB3 pathway downloaded from the NCI pathway interaction database (http://pid.nci.nih.gov). The roles and connections of the ErbB2, ErbB3 and Neuregulin proteins and peptides in supporting both the MEK and PI3K pathways were first mapped out around the year 2000 [30], and form the basis for the pathway displayed in Figure 4. The dependencies of RAF/MEK pathways [31], and PI3K/AKT pathways [32], on the levels of activated ErbB3 have been thoroughly demonstrated to show a very high level of reliance. In this network, the edges *e*_31_, *e*_32_, *e*_33_, *e*_36 _are transcription processes and are the only slow processes in the network; thus, they are assigned a "slow" label. A "fast" label, by default, is assigned to all other edges. Since no priority information is available in the database, no priority labels are assigned. The vector of observable states for this pathway is *y *= [FOS[n], CHRNA1[n], JUN[n], CHRNE[n]]*^T ^*, which are the output nodes of the transcription processes *e*_31_, *e*_32_, *e*_33_, and *e*_36_, respectively, whose activities can be measured by a microarray.

#### Dynamic trajectories

Assuming that the initial condition of the nodes marked with (*) is 1 and the others are 0, our algorithm gives 7 dynamic trajectories:

(20) $$\begin{array}{c} t_{1}=\left\{e_{19},e_{20},e_{21},e_{47}\right\}\in T\\ t_{2}=\left\{e_{19},e_{20},e_{21},e_{12},e_{47},e_{48}\right\}\in T\\ t_{3}=\left\{e_{19},e_{20},e_{21},e_{12},e_{13},e_{47},e_{48},e_{49}\right\}\in T\\ t_{4}=\left\{e_{19},e_{20},e_{21},e_{12},e_{13},e_{14},e_{47},e_{48},e_{49},e_{50}\right\}\in T\\ t_{5}=\left\{e_{19},e_{20},e_{21},e_{12},e_{13},e_{14},e_{47},e_{48},e_{49},e_{50},e_{51}\right\}\in T\\ t_{6}=\left\{e_{19},e_{24},e_{25},e_{26},e_{27},e_{28},e_{29},e_{30},e_{31},e_{32},e_{33}\right\}\in T\\ t_{7}=\left\{e_{1},e_{24},e_{25},e_{26},e_{27},e_{28},e_{29},e_{30},e_{31},e_{32},e_{33},e_{40},e_{19}\right\}\in T \end{array}$$

where the dynamic trajectories *t*_6 _and *t*_7 _contain transcription processes giving the final value of vector *y*, as *y *= [1, 1, 1, 0]^*T*^. The CPU time for computing these trajectories was 25.8 sec.

Drugs such as lapatinib can have a very strong inhibitory effect on levels of ErbB2/ErbB3 activity, though the drug's effects are themselves highly dependent on the presence of only low levels of ErbB2/ErbB3 [33]. The immunoblot figure in [33] shows that by suppressing activated ErbB2/ErbB3, both AKT and ERK phosphorylation will be turned off. To see how suppressing the activity of ErbB2/ErbB3 affects the network dynamics we consider another initial condition vector. Suppose that the nodes marked with (†) are initially set to 1 and the others are 0. For this initial condition, our algorithm yields no dynamic trajectory, *T *= ∅. This implies that none of the AKT and ERK phosphorylation processes occur, as we expected from the results in [33]. A similar dynamical analysis can be performed for other initial conditions, thereby giving the set of all possible outcomes.

## Current research

Each network edge in a dynamic trajectory can be mathematically expressed by a state transition matrix *A*. For instance, the transition from *x*_0 _to *x*_1 _in (4) can be described by

(21) $$x_{1}=A_{1}x_{0}$$

where

(22) $$A_{1}=\left[\begin{array}{ccc} 0 & 0 & 0\\ 1/2 & 0 & 1/2\\ 1/2 & 0 & 1/2 \end{array}\right]$$

A state transition matrix facilitates writing the network dynamics in terms of state space equations suitable for control system studies.

Given a network model *M *= (*N, E*), an initial condition *x*_0_, the calculated dynamic trajectories *T*, and recalling the notion of observable state vector *y*, one can mathematically describe the network dynamics corresponding to trajectory *t *∈ *T *by the following set of state space equations:

(23) $$\begin{array}{ccc} x_{k+1}= & A_{k+1}x_{k} & \\ y_{k}= & C\;x_{k} \end{array}$$

which is a linear discrete-time time-varying system. We alternatively refer to *y*_*k *_as the *system output *at the *k*-th time step.

From a biological viewpoint, not all dynamic trajectories are primarily of interest for design purposes, but those including observable states are important because the result of changes to network inputs or initial condition can be measured. Thereafter, a suitable experimental design can be proposed to attain a desired performance. We are currently conducting research on developing a new methodology to find logical relationships between network state variables and a set of desirable values for network observable states. This problem, referred to as a "backward inference problem", is a key step toward experimental design because it characterizes the necessary and sufficient conditions on the network state variables for which a desirable network performance can be obtained.

## Conclusions

We have proposed a new dynamical modeling methodology that can handle common biological knowledge without the need of exact parameters related to process dynamics. Our proposed approach has similarities in some aspects to other qualitative methods. The node update for conversion processes is similar to token passing in Petri nets, while the update for transcription processes functions like Boolean logic. These similarities occur because they are natural ways to describe the basic processes. However, to properly handle the pathway knowledge commonly available from a public database as a BioPAX file with minimal extra input, one needs to apply these canonical approaches in a practical way, which results in the kind of hybrid approach introduced in this paper. For instance, in the ErbB2/ErbB3 pathway example we were able to use different updating rules for the conversion and transcription processes that resulted in a dynamical model which captures the network dynamics more accurately. We would like to conclude by pointing out critical differences between our proposed method and the standard Petri net and Boolean network approaches.

First, for conversion type processes, a Petri net usually does not consider the role of activator by assuming they are always present, and the potential reversibility of the process, so that every physical entity ("place" in Petri net terminology) is either an input or an output. Although this can be partially solved by introducing a reading arc to indicate when a process can take place, it cannot be incorporated into the incidence matrix, which is critical for a fast update. As a result, a Petri net loses one of its most attractive properties. The use of an incidence matrix is further hampered by the synchronous update, which assumes that equal time elapses for each process.

For practical applications, a critical shortcoming of the standard approaches is the manner in which they handle uncertainty. A standard Petri net handles uncertainty in a random manner and Boolean networks demand that all uncertainties be resolved before simulation starts. In reality, a biological process often takes a deterministic fashion unknown to the modeler, which demands a comprehensive exploration of all the possibilities. Our choice of this hybrid approach is based on the purpose of utilizing public knowledge databases; however, the concept of exploiting all possible uncertainties is not limited to the specific procedure considered here. It can be applied to other qualitative methods, including both Boolean networks and Petri nets.

In conclusion, the entire procedure emulates how biologists actually utilize available pathway information. Our ultimate aim is to use predicted outcomes to design the most efficient experiments [34] to reduce uncertainty and find the best perturbation scheme to achieve desired network performance.

## Appendix

The input and output file formats of our software package are simple-text files. The input file contains the set of interactions/processes in the network and the initial condition vector in the following form:

$$\begin{array}{ll} \text{Process} & \left(process\#\right)\\ \text{Input} & \left(input\;nodes\right)\\ \text{Output} & \left(output\;nodes\right)\\ \text{Activator} & \left(activator\;nodes\right)\\ \text{Inhibitor} & \left(inhibitor\;nodes\right) \end{array}$$

For instance, for the following process

$$\begin{array}{ccc} & C & \\ & ↓ & \\ A+B & \to & D \end{array}$$

the input file contains:

$$\begin{array}{ccc} \text{Process} & 1 & \\ \text{Input} & \text{A} & \text{B}\\ \text{Output} & \text{D} & \\ \text{Activator} & \text{C} & \end{array}$$

The initial condition vector is added to the input file (after the list of processes) as follows:

$$\begin{array}{cc} \text{Entity} & \text{Value}\\ \left(node\right) & \left(initial\;condition\right) \end{array}$$

For the above process, if A, B and C are initially 1 and D is 0, then it will be added to the input file as:

$$\begin{array}{cc} \text{Entity} & \text{Value}\\ \text{A} & 1\\ \text{B} & 1\\ \text{C} & 1\\ \text{D} & 0 \end{array}$$

The output file summarizes the given initial condition vector and the calculated dynamic trajectories as well as the corresponding updates of the state vector in the following form:

Dynamic Trajectories for Initial Condition: *x*_0 _= *(given initial condition vector)*

Trajectory *t*_(*traj *#) _= {(*edge *#'*s*)}

State Vector Updates for *t*_(*traj *#)_:

Time Step 1: *(edge *#*)*

*x*_1 _= *(first update of the state vector)*

Time Step 2: *(edge *#*)*

*x*_2 _= *(second update of the state vector)*

...and so on for the next time steps and other dynamic trajectories. The output file for the above process and initial condition is:

Dynamic Trajectories for Initial Condition: *x*_0 _= [1, 1, 1, 0]^T^

Trajectory *t*_1 _= {*e*_1_}

State Vector Updates for *t*_1_:

Time Step 1: *e*_1_

*x*_1 _= [0, 0, 1, 1]^T^.

## Availability

A MATLAB software package enabled with Graphical User Interface (GUI) is programmed based on our proposed approach to perform dynamical modeling of uncertain biological networks. This package along with the real data examples presented in this manuscript are available at http://gsp.tamu.edu/Publications/supplementary/mohsenizadeh15a.

## Competing interests

The authors declare that they have no competing interests.

## Authors' contributions

D.N.M., J.H. and E.R.D. conceived the method. D.N.M. developed the dynamical modeling methodology and programmed the software package. D.N.M., J.H., M.B. and E.R.D. selected the real data examples and provided insights on the interpretation of the results. All authors wrote the manuscript.

## Supplementary Material

Additional file 1 **Dynamical Modeling Algorithm**. This file contains the detailed algorithm proposed for dynamical modeling of uncertain interaction-based biological networks. Click here for file
