# Supplementary material for: Dynamical modeling of uncertain interaction-based genomic networks
Source: BMC Bioinformatics. 2015 Sep 25;16(Suppl 13):S3. doi: 10.1186/1471-2105-16-S13-S3 (PMC4596957; doi:10.1186/1471-2105-16-S13-S3)
Supplement: Additional file 1 — Dynamical Modeling Algorithm. This file contains the detailed algorithm proposed for dynamical modeling of uncertain interaction-based biological networks. [file 1471-2105-16-S13-S3-S1.pdf]

Algorithm Dynamical Modeling Algorithm

Inputs: (N, E, x_0)
Outputs: (T, x)
$k \leftarrow 1$
$p, r \leftarrow 0$
$T, E_v, S_1', x_1 \leftarrow \emptyset$
$x_{in} \leftarrow x_0$
run Subroutine 1 (All Possible Edges): Inputs: (N, E, x_{in}), Outputs: E_v
if $E_v \neq \emptyset$ **then**
    $S_1' \leftarrow E_v$
    run Subroutine 2 (Apply Edge Properties): Inputs: (E_v, S_1'), Outputs: S_1'
    if $S_1' = \emptyset$ **then**
        $S_1' \leftarrow E_v$
    end if
    for $i = 1$, $i{+}{+}$, $i \leq$ number of edges in S_1' **do**
        $p \leftarrow p + 1$
        $x_{in} \leftarrow x_0$
        $e_c \leftarrow S_1'(i)$ (i-th edge in S_1')
        run Subroutine 3 (Update State Vector): Inputs: (N, E, x_{in}, e_c), Outputs: x_{out}
        $x_1^p \leftarrow x_{out}$
        $S_1^p \leftarrow \{S_1'(i)\}$
    end for
    while $p \neq 0$ **do**
        $k \leftarrow k + 1$
        $x_k \leftarrow \emptyset$
        $q \leftarrow p$, $p \leftarrow 0$
        for $i = 1$, $i{+}{+}$, $i \leq q$ **do**
            run Subroutine 4 (Cycle Detection): Inputs: S_{k-1}^i, Outputs: c
            if $c = 0$ **then**
                $E_v, S_k' \leftarrow \emptyset$
                $x_{in} \leftarrow x_{k-1}^i$
                run Subroutine 1 (All Possible Edges): Inputs: (N, E, x_{in}), Outputs: E_v
                if $E_v \neq \emptyset$ **then**
                    $S_k' \leftarrow E_v$
                    run Subroutine 2 (Apply Edge Properties): Inputs: (E_v, S_k'), Outputs: S_k'
                    if $S_k' = \emptyset$ **then**
                        $S_k' \leftarrow E_v$
                    end if
                    for $j = 1$, $j{+}{+}$, $j \leq$ number of edges in S_k' **do**
                        $p \leftarrow p + 1$
                        $x_{in} \leftarrow x_{k-1}^i$
                        $e_c \leftarrow S_k'(j)$ (j-th edge in S_k')
                        run Subroutine 3 (Update State Vector): Inputs: (N, E, x_{in}, e_c), Outputs: x_{out}
                        $x_k^p \leftarrow x_{out}$
                        $S_k^p \leftarrow \{S_{k-1}^i, S_k'(j)\}$
                    end for
                else if $S_{k-1}^i \notin T$ **then**
                    $r \leftarrow r + 1$
                    $t_r \leftarrow S_{k-1}^i$
                    $T \leftarrow \{T, \{t_r\}\}$
                end if
            else if $S_{k-1}^i \notin T$ **then**
                $r \leftarrow r + 1$
                $t_r \leftarrow S_{k-1}^i$
                $T \leftarrow \{T, \{t_r\}\}$
            end if
        end for
    end while
end if

Subroutine 1 (All Possible Edges)

Inputs: (N, E, x_{in})
Output: E_v

```
E_v ← ∅
for i = 1, i++, i ≤ number of nodes in N do
    n_i ← x_in(i)
end for
for i = 1, i++, i ≤ number of edges in E do
    c_inp, c_act, c_inh ← 0
    if number of input nodes of e_i ≠ 0 then
        for j = 1, j++, j ≤ number of input nodes of e_i do
            n_inp ← j-th input node of e_i
            if n_inp > 0 then
                c_inp ← c_inp + 1
            end if
        end for
        for j = 1, j++, j ≤ number of activator nodes of e_i do
            n_act ← j-th activator node of e_i
            if n_act > 0 then
                c_act ← c_act + 1
            end if
        end for
        for j = 1, j++, j ≤ number of inhibitor nodes of e_i do
            n_inh ← j-th inhibitor node of e_i
            if n_inh > 0 then
                c_inh ← c_inh + 1
            end if
        end for
        if c_inp = number of input nodes of e_i AND c_act = number of activator nodes of e_i AND c_inh = 0 then
            E_v ← {E_v, e_i}
        end if
    else
        for j = 1, j++, j ≤ number of activator nodes of e_i do
            n_act ← j-th activator node of e_i
            if n_act > 0 then
                c_act ← c_act + 1
            end if
        end for
        n_out ← output node of e_i
        if n_out = 0 AND c_act = number of activator nodes of e_i then
            E_v ← {E_v, e_i}
        end if
    end if
end for
```

Subroutine 2 (Apply Edge Properties)

Inputs: (E_v, S'_k)
Outputs: S'_k

```
for i = 1, i++, i ≤ number of edges in E_v do
    for j = 1, j++, j ≠ i, j ≤ number of edges in E_v do
        e_i ← i-th edge in E_v
        e_j ← j-th edge in E_v
        if e_i.Priority > e_j.Priority then
            S'_k ← S'_k − e_j
        end if
    end for
    if e_i.Speed = slow then
        S'_k ← S'_k − e_i
    end if
end for
```

Subroutine 3 (Update State Vector)

Inputs: (N, E, x_{in}, e_c)
Output: x_{out}

```
for $i = 1$, $i{+}{+}$, $i \leq$ number of nodes in $N$ do
    $n_i \leftarrow x_{in}(i)$
end for
if number of input nodes of $e_c \neq 0$ then
    for $j = 1$, $j{+}{+}$, $j \leq$ number of input nodes of $e_c$ do
        $n_{inp} \leftarrow j$-th input node of $e_c$
        $n_{inp} \leftarrow n_{inp} - 1$
    end for
    for $j = 1$, $j{+}{+}$, $j \leq$ number of output nodes of $e_c$ do
        $n_{out} \leftarrow j$-th output node of $e_c$
        $n_{out} \leftarrow n_{out} + 1$
    end for
else
    $c_{act} \leftarrow 0$
    for $i = 1$, $i{+}{+}$, $i \leq$ number of activator nodes of $e_c$ do
        $n_{act} \leftarrow i$-th activator node of $e_c$
        if $n_{act} > 0$ then
            $c_{act} \leftarrow c_{act} + 1$
        end if
    end for
    if $c_{act} =$ number of activator nodes of $e_c$ then
        $n_{out} \leftarrow$ output node of $e_c$
        $n_{out} \leftarrow 1$
    end if
end if
for $i = 1$, $i{+}{+}$, $i \leq$ number of edges in $E$ do
    if number of input nodes of $e_i = 0$ then
        $c_{act} \leftarrow 0$
        for $j = 1$, $j{+}{+}$, $j \leq$ number of activator nodes of $e_i$ do
            $n_{act} \leftarrow j$-th activator node of $e_i$
            if $n_{act} > 0$ then
                $c_{act} \leftarrow c_{act} + 1$
            end if
        end for
        if $c_{act} <$ number of activator nodes of $e_i$ then
            $n_{out} \leftarrow$ output node of $e_i$
            $n_{out} \leftarrow 0$
        end if
    end if
end for
for $i = 1$, $i{+}{+}$, $i \leq$ number of nodes in $N$ do
    $x_{out}(i) \leftarrow n_i$
end for
```

Subroutine 4 (Cycle Detection)

Inputs: S
Outputs: c

```
$c \leftarrow 0$
$l \leftarrow 0$
$m \leftarrow$ (number of edges in $S)/2$
if $m$ is an integer then
    for $i = 1$, $i{+}{+}$, $i \leq m$ do
        if $S(i) = S(i + m)$ then
            $l \leftarrow l + 1$
        end if
    end for
    if $l = m$ then
        $c \leftarrow 1$
    end if
end if
```
